# Integrative functional analyses using rainbow trout selected for tolerance to plant diets reveal nutrigenomic signatures for soy utilization without the concurrence of enteritis

**DOI:** 10.1371/journal.pone.0180972

**Published:** 2017-07-19

**Authors:** Jason Abernathy, Andreas Brezas, Kevin R. Snekvik, Ronald W. Hardy, Ken Overturf

**Affiliations:** 1 Hagerman Fish Culture Experiment Station, USDA-ARS, Hagerman, Idaho, United States of America; 2 Aquaculture Research Institute, University of Idaho, Hagerman, Idaho, United States of America; 3 Department of Veterinary Microbiology and Pathology, Washington State University, Pullman, Washington, United States of America; Ohio State University, UNITED STATES

## Abstract

Finding suitable alternative protein sources for diets of carnivorous fish species remains a major concern for sustainable aquaculture. Through genetic selection, we created a strain of rainbow trout that outperforms parental lines in utilizing an all-plant protein diet and does not develop enteritis in the distal intestine, as is typical with salmonids on long-term plant protein-based feeds. By incorporating this strain into functional analyses, we set out to determine which genes are critical to plant protein utilization in the absence of gut inflammation. After a 12-week feeding trial with our selected strain and a control trout strain fed either a fishmeal-based diet or an all-plant protein diet, high-throughput RNA sequencing was completed on both liver and muscle tissues. Differential gene expression analyses, weighted correlation network analyses and further functional characterization were performed. A strain-by-diet design revealed differential expression ranging from a few dozen to over one thousand genes among the various comparisons and tissues. Major gene ontology groups identified between comparisons included those encompassing central, intermediary and foreign molecule metabolism, associated biosynthetic pathways as well as immunity. A systems approach indicated that genes involved in purine metabolism were highly perturbed. Systems analysis among the tissues tested further suggests the interplay between selection for growth, dietary utilization and protein tolerance may also have implications for nonspecific immunity. By combining data from differential gene expression and co-expression networks using selected trout, along with ontology and pathway analyses, a set of 63 candidate genes for plant diet tolerance was found. Risk loci in human inflammatory bowel diseases were also found in our datasets, indicating rainbow trout selected for plant-diet tolerance may have added utility as a potential biomedical model.

## Introduction

Manufactured from wild-catch marine forage fish and byproducts from fish processing, fishmeal (FM) is a primary source of protein in aquaculture feeds (aquafeeds). Since annual FM production is already fully utilized, mainly in aquafeeds, finding replacements for FM has been a concern for decades. A growing concern in recent years is the rising cost, as global demand for salmonid and other marine foods has increased while, over the same period, global production of FM has been in decline. Costs notwithstanding, the amount of marine-produced protein needed to feed expanding global aquaculture production systems is not available and is a barrier to the long-term growth of the industry. The decrease in availability of FM along with the increase in costs has led to international efforts toward reformulation of aquafeeds with higher percentages of plant-based products, replacing ever-decreasing amounts of marine protein with ever-increasing amounts from plant-protein sources including plant-meal (PM) and plant-protein concentrates. Supply-and-demand along with the push toward more sustainable aquaculture are not the only concerns with such changes however, as biological barriers to the reformulation of aquafeeds also exist in attempts to reduce and replace FM. Main barriers include incomplete information on nutritional requirements of major farmed species, differences in the digestibility and bioavailability of essential nutrients in reformulated feeds, the presence of antinutritional factors in plant feedstuffs, and the potential for a reduction in palatability and ingestion of aquafeeds with lower percentages of fish-derived ingredients [1–7].

Aquafeeds containing high amounts of plant-based proteins can have an array of effects on fish, starting with reduced feed intake due to palatability that lowers weight gain. Other effects take longer to manifest and include lower feed efficiency, altered gut microflora, immune stimulation and/or intestinal dysfunction [8]. Total replacement of FM with plant proteins has been shown repeatedly to reduce growth in salmonids and other marine species [9–14]. However, suboptimal growth and/or reduced protein retention efficiency results even when fish are fed partial replacement diets and when all known essential nutrients, including amino acids, are present in the diet above required levels [15–18]. Salmonid aquaculture faces a related challenge in the substitution of FM with PM in feeds in that some plant ingredients, such as soya and other legumes, induce severe inflammation, or enteritis, in the distal intestine when present in the diet above certain levels (reviewed in [8]). Numerous studies have described the physiological responses and/or deleterious effects of enteritis related to FM-to-PM diet substitution in fish [19–26].

The United States Department of Agriculture—Agricultural Research Service (USDA-ARS) has been selecting trout based on growth performance when fed an all-plant protein feed for 16 years (eight generations). The line was created by introgression of nine domesticated commercial and conservation stocks. The selected trout (designated ARS-KO strain) grows twice as rapidly as parental lines and does so when fed a high-soy, all plant-protein feed [27]. Further, the selected strain does not develop distal intestine enteritis [28]. This trait makes it possible for the selected strain to be a “positive-control” to compare with non-selected trout strains that develop enteritis when fed plant-protein feeds.

In this report, a non-selected strain (House Creek; HC) and selected (ARS-KO) rainbow trout were fed two experimental feeds over a 12-week period—either a fishmeal-based feed similar to a commercial trout feed formulation or a diet in which all protein was supplied from plant-derived sources, including a high percentage of soy-based proteins. Since dietary alterations cause changes in central metabolism and as growth was the main trait used in the selection program, global gene expression changes in liver and muscle tissue between strains fed with different diets were assessed. Through the combination of differential gene expression with weighted network analyses, using the enteritis-free (selected) trout strain as a control, expression signatures and mechanistic genes were revealed that may be useful marker candidates for surveillance, manipulation and/or selection toward the plant-diet tolerance trait.

## Materials and methods

### Ethics statement

Use of animals in this study was approved by the University of Idaho Institutional Animal Care and Use Committee (IACUC) under the protocol number 2013098. Fish were euthanized by an overdose of tricaine methanesulfonate prior to dissections.

### Rainbow trout husbandry and sampling

**Fig 1:**
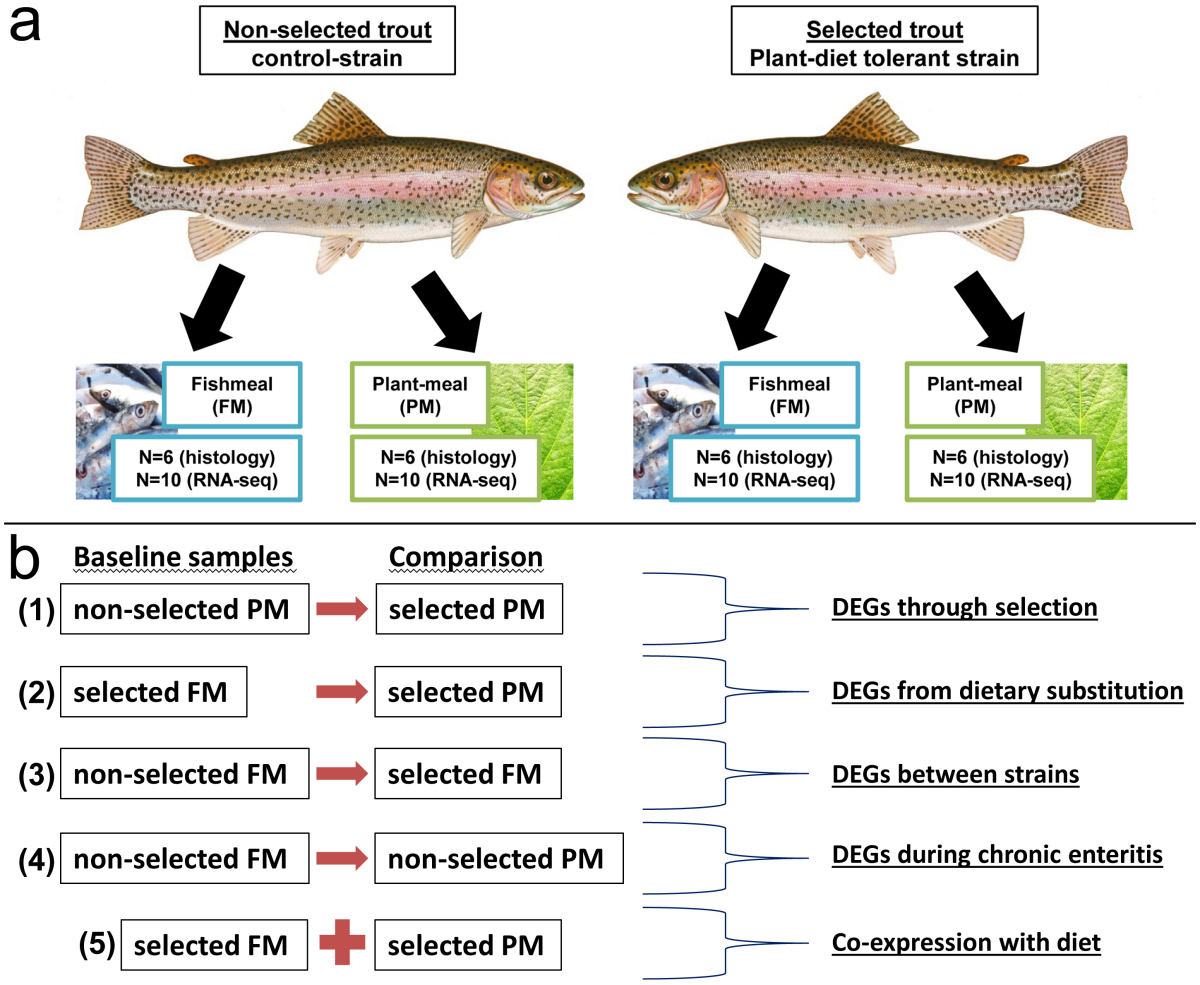
Experimental design.

HC (non-selected) and ARS-KO (selected) strains [27] were maintained on either a FM or PM based-diet for 12-weeks in triplicate 144 liter tanks. The fish were fed commercial trout fry food (52% protein and 16% lipid; Skretting USA, Tooele, UT) from first feeding until 5 g average weight. At 5 g the fish strains were split into the following replicate treatments (3 tanks of 150 fish each of ARS-KO fed the FM diet, 3 tanks of ARS-KO fed the PM diet, 3 tanks of HC fed the FM diet, and 3 tanks of HC fed the PM diet). Diet formulations are shown in Table 1. In a previous study, we found enteritis is evident in non-selected trout fed a PM diet after 12-weeks of feeding [28]. After 12-weeks’ of feeding, trout were randomly selected from tanks, euthanized by an overdose of tricaine methanesulfonate (MS-222; 250 mg/mL; buffered with sodium bicarbonate), and tissues were excised and either fixed for histological examination or flash-frozen in liquid nitrogen and stored at -80°C for RNA extraction. A total of 24 samples were processed for histology: six HC fish and six ARS-KO fish on PM diet, six HC fish and six ARS-KO fish on FM diet, sampling distal intestine from each individual. A total of 80 samples were processed for RNA: 10 HC fish and 10 ARS-KO fish on PM diet, 10 HC fish and 10 ARS-KO fish on FM diet, sampling two tissues (liver and muscle) per individual. A diagram of the experimental design is provided in Fig 1a. As we are presenting a 2x2 factorial design, four different comparisons (termed “selection”, “diet”, “strain” and “enteritis” effects herein) will be discussed throughout and are detailed in Fig 1b.

**Table 1:** Formulations of experimental diets.

INGREDIENT (% of total)	Plant Protein	Fishmeal
	PM diet	FM diet
Anchovy meal	-	41.71
Krill meal	-	28
Soy protein concentrate	29.98	-
Soybean meal	20.00	-
Wheat gluten meal	10.04	-
Wheat flour	15.00	15.4
Fish oil	5.00	11.8
Soybean oil	8.43	-
Vitamin C	-	0.36
Choline	0.50	0.6
TM salt	0.50	0.1
Vitamin premix	0.80	1
Corn gluten, yellow	3.75	-
Dicalcium phosphate	2.55	-
Bioplex mineral supplement	1	1
Lys	1.47	-
Met	0.45	-
Taurine	0.50	-
Astaxanthin	0.03	0.03

### Histology

Distal intestine (DI) is delimited proximally by the first complex fold and distally by the anal opening. For each individual, a 5 mm section of DI, excised immediately distal to the ileo-rectal valve, was sampled and fixed in phosphate buffered formalin (Fisher Scientific, Waltham, MA) for 24 h before being transferred and held in 70% ethanol until processing.

All DI tissue samples were ultimately fixed in buffered formalin, dehydrated in ethanol, equilibrated in xylene and embedded in paraffin (Tissue Tek #4005, melting point: 56°C) for imaging. For each sample, 3 μm sections were cut in the longitudinal plane and stained with hematoxylin and eosin (H&E). Slides were visually inspected using light microscopy (Zeiss AxioScope A1 light microscope, Carl Zeiss Ltd, Cambridge, UK) in a random fashion and with the evaluator blinded to treatment. DI tissue sections were scored according to the following variables on a continuous analogue scale, similar as was previously described [29]: Mucosal fold fusion (MF), lamina propria width and cellularity (LP), sub-epithelial mucosa width and cellularity (SM), degree of enterocyte supranuclear vacuolization (SNV), number of goblet cells (GC), number of eosinophilic granulocytes (EG), and total inflammatory cell number (TIC). The score range was arbitrarily set from one to five for each variable, with higher numbering indicative of increasing severity, to obtain a cumulative histopathology score. Differences between scores were assessed by nonparametric Kruskal-Wallis test and variables within scores were further assessed by Dunn’s post-hoc test.

### RNA sequencing

Total RNA was isolated from tissues using the TRIzol reagent according to the manufacturer’s protocol (ThermoFisher Scientific, Waltham, MA). Total RNA from each sample was then assessed for quantity on a spectrophotometer (NanoDrop 2000, ThermoFisher Scientific) and quality using the Agilent BioAnalyzer with the RNA 6000 Nano Kit (Santa Clara, CA). RNA Integrity numbers (RIN) > 8 were considered high-quality for processing the RNA further to be sequenced. All samples met this criterion.

Total RNA samples were normalized to 1 μg and treated for potential DNA contamination using Amplification Grade DNase I (ThermoFisher Scientific) according to the supplied protocol. Samples were precipitated at least overnight with 3 M sodium acetate-ethanol and reconstituted in 28 μL nuclease-free water. Samples were then processed for removal of both cytoplasmic and mitochondrial ribosomal RNA (rRNA) using the Ribo-Zero Gold rRNA Removal Kit (Illumina, San Diego, CA) per the protocol from the manufacturer. We found that this kit, based on mammalian rRNA probes, is conserved and works well to remove a majority of trout contaminating rRNAs [30]. Ribosomal RNA-reduced samples were then made into directional RNA-sequencing libraries for Illumina HiSeq sequencing using the ScriptSeq v2 RNA-Seq Library Preparation Kit (Illumina) per the instructions, with one modification: we used ½ input RNA volume and ½ reagents to make the libraries. During the PCR amplification step, each sample was barcoded using TruSeq indexes #1–12 (Illumina). Libraries were assessed for quality per the ScriptSeq protocol by using the Agilent BioAnalyzer with the High Sensitivity DNA Kit. Libraries were quantified by qPCR on a Real-Time PCR Instrument (QuantStudio 6 Flex, ThermoFisher Scientific) using a standard curve. Equimolar amounts of samples with different indexes were pooled in multiples of 8 (4 muscle and 4 liver). Pooled libraries were sent for 100 bp single-read sequencing on an Illumina HiSeq 1500. The RNA sequencing datasets are available in the NCBI Gene Expression Omnibus (GEO) repository under accession number GSE79531.

### Bioinformatics

Base calling, generation of quality-scores, demultiplexing and generation of raw data into FASTQ files was either performed in the Illumina sequencer or using the native CASAVA software. Raw reads were then processed for quality control (QC) using the Trim Galore! software. The default parameters were used in this software for both Illumina adapter removal and for trimming of low-quality ends from reads, with Phred Q20 as quality score cut-off. Sequences after QC were filtered for potential ribosomal RNA contamination using the Bowtie2 software [31]. All *Oncorhynchus* species rRNAs available in GenBank were used to align against, where unaligned reads were output for further usage. The default Bowtie2 parameters were used for rRNA screening. Bowtie2 was then used for alignment of reads to the rainbow trout transcriptome [32] with the alignment parameters as recommended by the eXpress software [33]. Bowtie2 alignments were streamed into the eXpress software to generate read-counts and FPKM (Fragments Per Kilobase of transcript per Million mapped reads) values, using the recommended parameters but also including strand information. Effective read counts output from the eXpress software were used to calculate gene expression fold-changes in the DESeq2 package [34] of R-bioconductor, with significant differences assessed by p-values adjusted for multiple comparisons (p-adj < 0.05). For highest confidence, transcripts with an FPKM < 1 were lastly removed from the final data [35].

For Gene Ontology (GO) enrichment analyses, published rainbow trout transcripts [32] were uploaded into the BLAST2GO program [36]. Transcripts were functionally annotated by BLASTx (E-value < 1E-3) searches to the non-redundant (*nr*) protein sequence database at the NCBI and mapped to GO terms, Enzyme Codes (EC) numbers and/or InterPro IDs, where available. This list provided a “reference set” for enrichment analyses. Significantly regulated (p-adj < 0.05; FPKM > 1) differentially expressed (DE) gene lists between each comparison were used as a “test set” for enrichment analysis to the “reference set” by two-sided Fisher’s Exact Test. Enrichment of all the three major GO categories (Biological Process (BP), Molecular Function (MF) and Cellular Component (CC)) was determined. Data was considered statistically significant using a False Discover Rate (FDR) of FDR < 5%. Where possible, data characterized in BLAST2GO were also mapped to Kyoto Encyclopedia of Genes and Genomes (KEGG) pathways using the software.

A weighted correlation network analysis (WGCNA) was performed to determine a consensus gene co-expression network among all ARS-KO (selected) individuals, including those on FM (n = 10) and those on PM (n = 10) diets. Information included for the network build includes liver RNA-seq data, total fish weight, total liver weight and hepatosomatic index (HSI) for each individual (S1 Table). In the absence of the development of distal intestine enteritis in these fish given either diet (i.e. without disease as a confounder), we are as a result parsing out those expression signatures most important in the central (liver) metabolism of dietary replacement by incorporating the selected fish on a PM diet as a positive control. For the analysis, the WGCNA package in R-bioconductor was used [37]. Per the author’s suggestions using RNA-seq data, filtered-transformed FPKM values (S2 Table) were used in the construction instead of read-counts. First, FPKM values were filtered, where any row (gene) was removed when the average of more than 18 of the samples (≥ 90%) had an FPKM value < 1. The data was then log-transformed [log_2_(FPKM +1)] for variance stabilization. These data were then subjected to the WGCNA package per the vignette. Briefly, individuals were first assessed for outliers by clustering RNA-seq data with trait data (none were found / removed). Next, soft thresholding was assessed using a signed hybrid network type. Based on the analysis, a soft threshold (β = 8) was selected and then gene clustering based on topological overlap measure of dissimilarity was performed. To ultimately detect modules of co-expressed genes, a minimum module size of 30 was set as was a cut-height threshold of MEDissThres = 0.20, thereby merging highly correlated (*r* > 0.80) eigengenes and then calculating significant associations with the trait of interest (diet). As is standard in WGCNA, modules will be referred to by their color. GO analysis on genes within each significant module was performed as described above.

For reporting of data, trout transcripts were mapped to their most plausible homolog by BLASTx of the *nr* database in GenBank then accession numbers were converted to *Homo sapiens* gene symbols using bioDBnet [38], where available. Gene IDs throughout are based on the letter/numbering from the FASTA file header of the published rainbow trout transcriptome [32].

### Validation of RNA-sequencing by quantitative PCR

We selected a set of genes for validation with reverse-transcription quantitative PCR (RT-qPCR) using custom TaqMan Gene Expression Assays (ThermoFisher Scientific). A total of eight TaqMan RT-qPCR assays were performed on liver RNA: one up-regulated and one down-regulated gene of interest per each of the four comparisons. Genes of interest selected include: betaine-homocysteine S-methyltransferase 1 (BHMT1); microfibril-associated glycoprotein 4 (MFAP4); GSK-3-binding protein (GSK3); AP-3 complex subunit beta-2 (AP3B2); early growth response 1 (EGR1); elastase-1 (ELA1); mevalonate kinase (MVK); interleukin-17 receptor E-like (IL17REL). TaqMan assays were designed using Primer3 [39], NCBI Primer-BLAST [40] and/or the native Custom TaqMan Assay Design Tool at the manufacturer’s website (ThermoFisher Scientific) using sequences from the rainbow trout transcriptome [32] as reference.

For RT-qPCR reactions, total RNA isolated from the same rainbow trout individuals and tissue as for RNA-seq was used. For each sample, total RNA was normalized to 1 μg, DNase-treated and then salt-ethanol precipitated. DNA-free total RNA was reconstituted and normalized to a concentration of 15 ng/μL. RT-qPCR assays were performed in 384-well format using a total volume of 15 μL, including 5 μL of normalized RNA and 10 μL master mix per well in one-step reactions, combining the Verso 1-step RT-qPCR Mix with ROX kit (ThermoFisher Scientific) components with TaqMan assays. Each well of the master mix includes the following: 7.5 μL of 1-Step qPCR-ROX Mix (2X); 0.12 μL of Verso Enzyme Mix; 0.625 μL of TaqMan probe/primers assay; and 1.755 μL nuclease-free water.

RT-qPCR runs were performed on the QuantStudio 6 Flex System (ThermoFisher Scientific) using the following cycling parameters: 50°C for 30 min and then 95°C for 15 min, followed by 40 cycles of 95°C for 15 s and 60°C for 60 s. Between each comparison, all individual liver samples (biological replicates) used for RNA-seq were also tested by RT-qPCR. Technical replicates were performed in duplicate. No-template control reactions, housekeeping gene amplifications as well as a standard curve were included in each run. Data output was assessed manually for quality against the standard curve and for low standard deviation. Cycle threshold (Ct) values were collected. Relative quantification (fold-change) was calculated by normalizing the gene of interest to the housekeeping gene using the comparative Ct method [41] in the Relative Expression Software Tool (Qiagen, Valencia, CA), with standard curve adjustment for amplification efficiency [42]. Both 18S rRNA and β-actin genes were amplified on each run as housekeeping genes; the more stable of the two, assessed as lowest standard deviation between replicates, was used for ΔCt calculations. Primer information is provided in S3 Table.

## Results

### Histology

**Fig 2:**
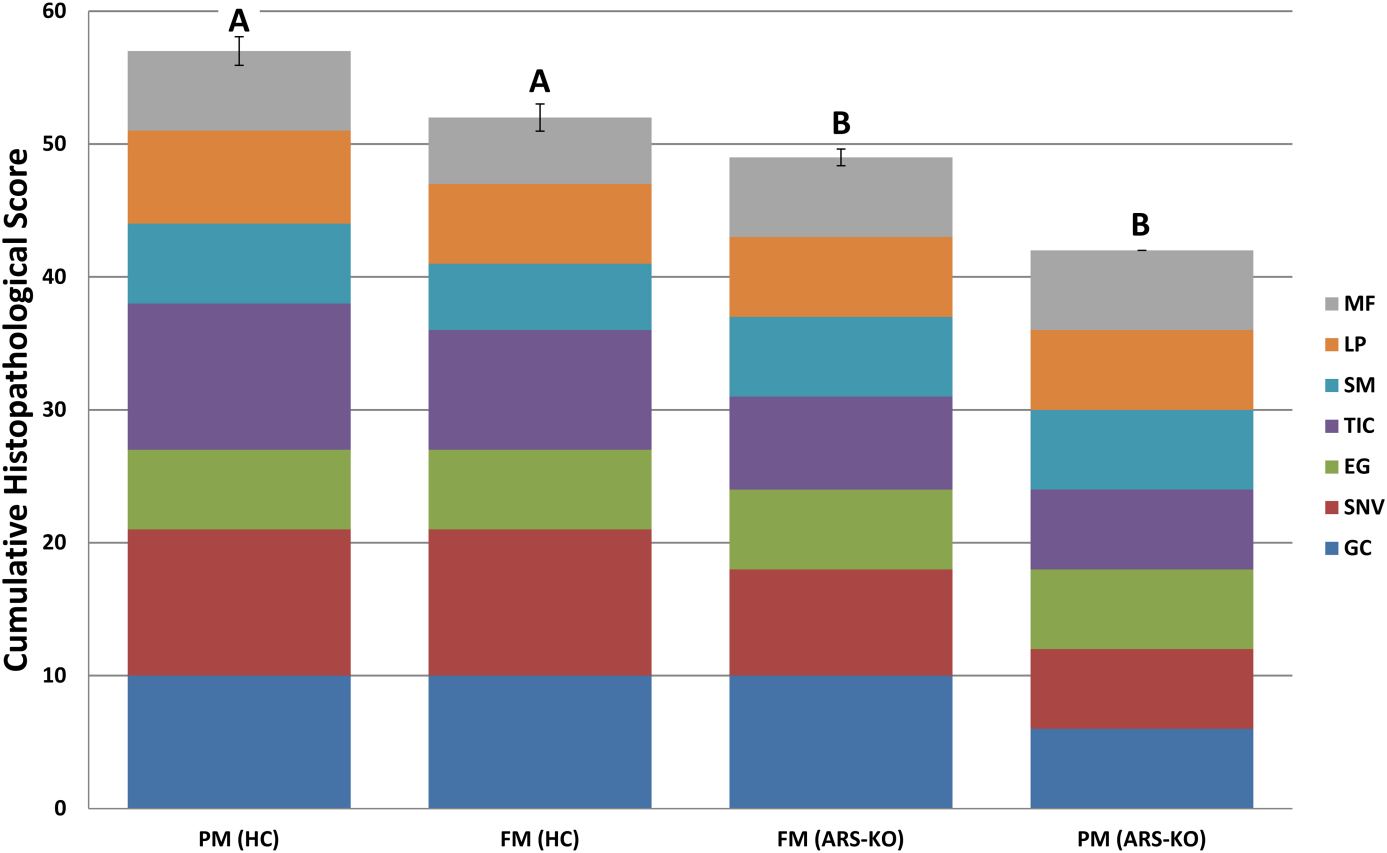
Bar chart of histopathological scoring. The Y-axis shows cumulative score, where higher scoring equals increase in severity. The X-axis indicates fish strain (HC or ARS-KO) along with treatment (PM or FM diet). The chart key and color are indicative of scoring variables: Mucosal fold fusion (MF), lamina propria width and cellularity (LP), sub-epithelial mucosa width and cellularity (SM), total inflammatory cell number (TIC), number of eosinophilic granulocytes (EG), degree of enterocyte supranuclear vacuolization (SNV) and number of goblet cells (GC).

**Fig 3:**
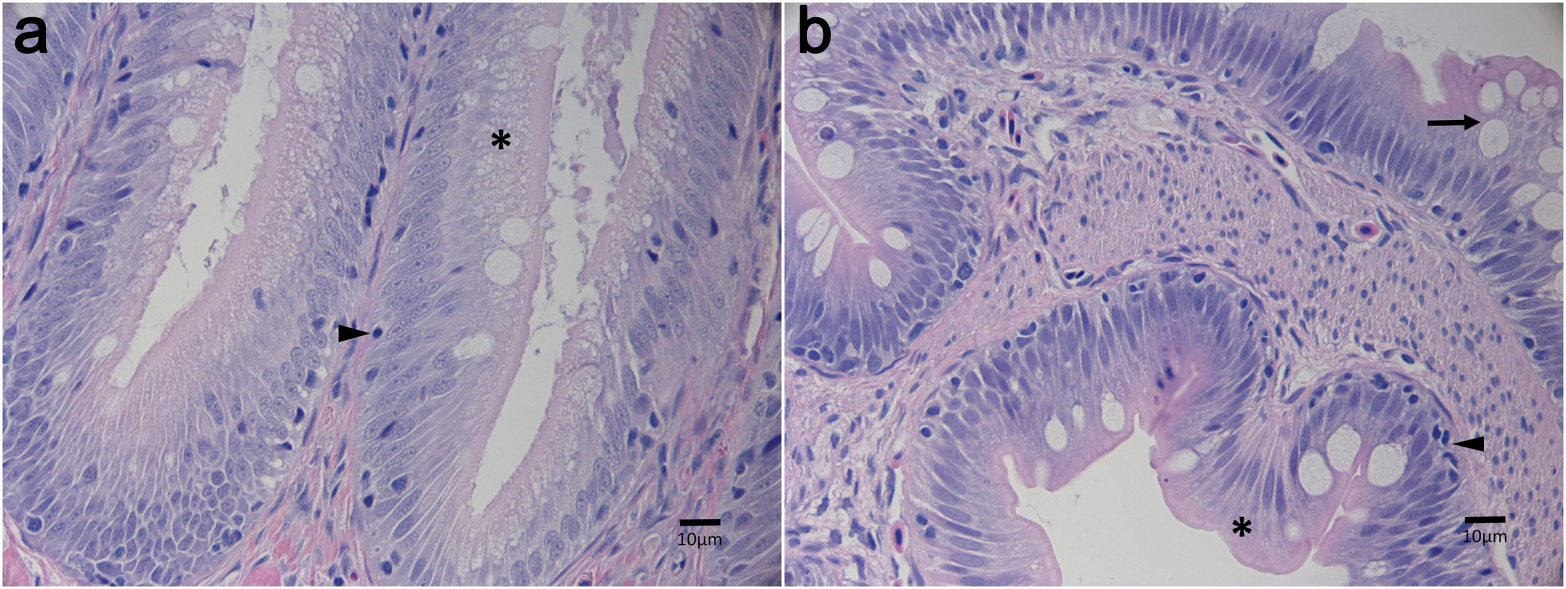
Photomicrographs of distal intestines at 400x magnification. (a). A representative image of selected fish reared for 12-weeks on plant protein based feed, similar to selected and non-selected fish reared on the fishmeal based feed (b). A representative image of non-selected fish reared for 12-weeks on the plant protein based feed. Intracytoplasmic supranuclear vacuoles (asterisk), intraepithelial lymphocytes (arrowhead) and mucous cell hyperplasia (arrow) are so indicated. Scale is depicted by horizontal bar.

The results of the histological evaluation from the sampling are presented in Fig 2. The highest histopathological score, indicative of the largest amount of observed tissue disruption and inflammation, was seen in the non-selected (HC) fish fed the PM diet, followed by HC fed the FM diet, selected (ARS-KO) fish fed the FM diet and ARS-KO fish fed the PM diet. There were significant differences (p = 5.7e^-5^) between the selected and non-selected fish. The differences between treatments was largely accounted for by supranuclear vacuolization (p = 0.005) and total inflammatory cells (p = 0.09) present. As seen in Fig 3a, DI in a selected fish fed the PM diet display well-organized mucosal epithelial cells, contains normal intracytoplasmic supranuclear vacuoles and rare intraepithelial lymphocytes. This is in contrast with a non-selected fish fed the PM diet (Fig 3b), whose DI displays slightly disorganized mucosal epithelial cells, with reduced numbers of intracytoplasmic supranuclear vacuoles and increased numbers of intraepithelial lymphocytes along with mucous cell hyperplasia. Overall, DI tissue of fish in the selected PM treatment appeared structurally similar to those of the non-selected FM and selected FM treatments (Fig 3a), whereas DI of fish in the non-selected PM treatment (Fig 3b) showed higher signs of inflammation under microscopic examination, evident as TIC and SNV (Fig 2).

### RNA-sequencing summary and validation

A total of 80 RNA-seq libraries were prepared and sequenced, including 40 muscle and 40 liver samples from individual trout based on the design schema (Fig 1). The overall average sequencing depth was > 30 M reads per sample. After QC, the composite of this dataset produced over two billion high-quality, rRNA-free reads from which to draw conclusions for gene expression analyses. This high-quality dataset presented an average of 25.4 M reads per sample. Sequencing using a tissue-swap design, where equal numbers of each tissue were sequenced together, provided for a consistent number of QC-reads between tissues, averaging 26.1 M and 24.7 M reads per muscle and liver sample, respectively. Alignment of the data to the 44990 transcripts from the published rainbow trout transcriptome [32] gave an average mapping rate of 41.8% for muscle samples and 42.7% for liver samples, for an approximate overall mapping rate of 42%.

As this is the first study of its kind using comparisons with these trout strains and treatments, we wanted to validate the results of the RNA-seq analysis using quantitative PCR. We designed custom TaqMan assays for two interesting genes of each comparison, one up- and one down-regulated. We used the same total RNA isolated for RNA-seq as template for qPCR. As shown in Table 2, all genes were validated by qPCR. We observed that the magnitude in fold-change is similar and the fold-change directions (±) are the same between technologies.

**Table 2:** Quantitative PCR (RT-qPCR) validation of RNA-seq using liver samples from each of the four comparisons, illustrated in Fig 1.

Baseline / reference samples	Δ samples	Gene	Gene ID	Fold-change (RNA-seq)	p-adj value (RNA-seq)	Fold-change (qPCR)	p-value (qPCR)	major effect assessed
non-selected PM	selected PM	MFAP4	C15301_c1_seq1	2.84	<0.001	2.16	0.001	selection
		BHMT1	C8153_c0_seq1	-2.94	<0.001	-4.90	<0.001	
selected FM	selected PM	EGR1	C27383_c0_seq1	3.40	<0.001	3.24	0.002	dietary
		ELA1	C494_c0_seq1	-4.95	<0.001	-2.24	0.003	
non-selected FM	selected FM	GSK3	C18587_c0_seq1	3.85	<0.001	2.69	<0.001	strain
		AP3B2	C40330_c0_seq1	-2.86	<0.001	-2.18	0.003	
non-selected FM	non-selected PM	MVK	C91938_c0_seq1	1.99	<0.001	1.91	0.001	enteritis
		IL17REL	C172972_c0_seq1	-2.52	<0.001	-5.62	<0.001	

### Differential gene expression

As outlined in Fig 1b, we evaluated differential gene expression as follows. First, by comparing the non-selected stain to the selected strain, both fed the PM diet, we were able to assess the effects of selection on DE of genes on a given diet. Second, using the selected strain as a positive-control, we assessed DE of genes associated with the physiological effects of diet substitution, since we are doing so in the absence of enteritis. Third, comparing the non-selected strain to the selected strain fed the FM diet, we assessed the basal gene expression differences between the strains under a control-like treatment, here a typical fish-protein based diet. Finally, using only the non-selected strain fed different diets we assessed enteritis development after 12-weeks of feeding.

**Fig 4:**
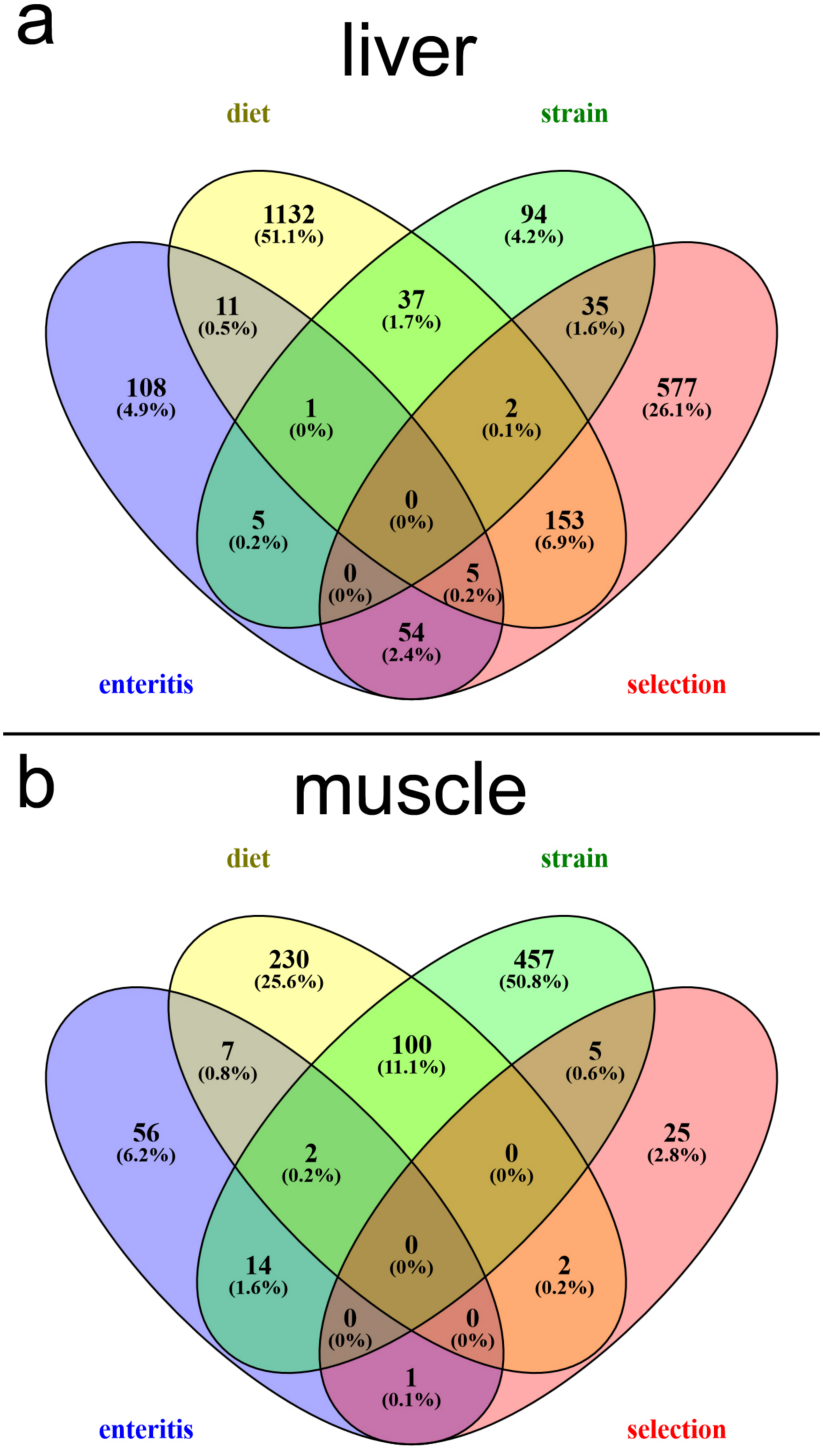
Venn diagram of the number of significant (p-adj < 0.05; FPKM > 1) differentially expressed genes.

In liver tissues (S4–S7 Tables) we found 826 DEGs that satisfied our stringency (p-adj < 0.05; FPKM > 1) in the selection comparison, assessing the differences between non-selected and selected fish fed the PM diet (Fig 1b). This included 410 down-regulated genes and 416 up-regulated genes. For the diet comparison, 1341 DEGs were identified, including 693 down-regulated and 648 up-regulated genes. For the strain comparison, 174 DEGs were identified, including 100 down-regulated and 74 up-regulated genes. For the enteritis group comparisons in liver tissues, 184 DEGs were identified, including 75 down-regulated and 109 up-regulated genes. DEGs between comparisons in liver tissues are visualized in a Venn diagram (Fig 4a). The greatest overlap in DEGs occurred between diet-selection with 153 DEGs contributing 6.9% to the Venn. Next was enteritis-selection, where 54 DEGs contributing 2.4% to the Venn overlapped, followed by 37 DEGs between the diet-strain comparison. Few DEGs had multiple overlap and no common genes were differentially expressed among all four groups.

In muscle tissues (S8–S11 Tables) we found 33 DEGs in the selection comparison, assessing the differences between non-selected and selected fish fed the PM diet (Fig 4b). This included 20 down-regulated genes and 13 up-regulated genes. For the diet comparison, 341 DEGs were identified, including 190 down-regulated and 151 up-regulated genes. For the strain comparison, 578 DEGs were identified, including 288 down-regulated and 290 up-regulated genes. For the enteritis group comparisons in liver tissues, 80 DEGs were identified, including 15 down-regulated and 65 up-regulated genes. DEGs between comparisons in muscle tissues are visualized in a Venn diagram (Fig 4b). By far the greatest overlap in DEGs occurred between diet-strain comparisons with 100 DEGs contributing 11.1% to the Venn. Next was enteritis-strain, where 14 DEGs contributing 1.6% to the Venn overlapped. Similar to liver comparisons, few DEGs had multiple overlap and no common genes were differentially expressed among all four groups.

Gene ontology analyses were also performed on DEGs in liver and muscle tissues. In liver, a total of 168 GO groups were found to be significant (S12 Table). This included 34 CC, 52 MF and 82 BP categories. By far the most ontologies were identified underlying the diet design / comparison, contributing 100 differentially represented groups spanning all three categories, followed by selection, enteritis and lastly strain, which had only one significantly enriched category, having oxidoreductase activity over-represented. As clearly liver tissue had more DEGs and also differentially regulated ontologies than did muscle, the top five most significant (lowest FDR) GOs are represented in the main text (Table 3). The same pattern holds true as with the full dataset (S12 Table). We see that in the diet design, all GOs from all three categories are over-represented when compared to all genes in the trout transcriptome. A similar pattern exists for the enteritis design, except that no GOs from the MF category were found to be significant. The selection design had the lone significant under-represented group: a cellular component GO representing plasma membrane (Table 3).

**Table 3:** Gene ontology enrichment in liver. The top five (where available; based on lowest FDR) most significant descriptions in each category within each design are shown. A complete listing is provided in S12 Table.

Design	Category	Representation	GO-ID	Description
diet	CC	OVER	GO:0022625	cytosolic large ribosomal subunit
			GO:0022627	cytosolic small ribosomal subunit
			GO:0032040	small-subunit processome
			GO:0005789	endoplasmic reticulum membrane
			GO:0070062	extracellular exosome
	MF		GO:0003735	structural constituent of ribosome
			GO:0044822	poly(A) RNA binding
			GO:0019843	rRNA binding
			GO:0003924	GTPase activity
			GO:0046933	proton-transporting ATP synthase activity, rotational mechanism
	BP		GO:0006614	SRP-dependent cotranslational protein targeting to membrane
			GO:0015986	ATP synthesis coupled proton transport
			GO:0006415	translational termination
			GO:0019083	viral transcription
			GO:0000184	nuclear-transcribed mRNA catabolic process, nonsense-mediated decay
enteritis	CC	OVER	GO:0044429	mitochondrial part
			GO:0005740	mitochondrial envelope
			GO:0031967	organelle envelope
			GO:0031975	envelope
			GO:0031966	mitochondrial membrane
	BP		GO:0008299	isoprenoid biosynthetic process
			GO:0008610	lipid biosynthetic process
			GO:0006694	steroid biosynthetic process
			GO:0006720	isoprenoid metabolic process
			GO:0044711	single-organism biosynthetic process
selection	CC	OVER	GO:0000276	mitochondrial proton-transporting ATP synthase complex, factor F(o)
			GO:0005747	mitochondrial respiratory chain complex I
			GO:0070069	cytochrome complex
		UNDER	GO:0005886	plasma membrane
	MF	OVER	GO:0009055	electron carrier activity
			GO:0015078	hydrogen ion transmembrane transporter activity
			GO:0030976	thiamine pyrophosphate binding
			GO:0004591	oxoglutarate dehydrogenase (succinyl-transferring) activity
			GO:0008137	NADH dehydrogenase (ubiquinone) activity
	BP		GO:0046496	nicotinamide nucleotide metabolic process
			GO:0006956	complement activation
			GO:0006123	mitochondrial electron transport, cytochrome c to oxygen
			GO:0015976	carbon utilization
			GO:0006108	malate metabolic process
strain	MF	OVER	GO:0016491	oxidoreductase activity

GO analysis based on muscle DEGs revealed 37 GO-IDs that were significant for enrichment among the datasets (Table 4). The diet and strain designs had the largest numbers of differentially represented GOs, both at 17. The enteritis and selection design / comparisons had 2 and 1 GOs, respectively. Within the diet design, all three GO categories were identified and each had both over- and under-representation. A similar arrangement was found in the strain design, with the exception that no GOs within the CC category were identified as under-represented. Two BP GOs were found over-represented in the enteritis group comparison, metabolic processes with folic-acid and/or pteridine-containing compounds. A single MF GO was identified a significantly over-represented in the selection comparison: endoplasmic reticulum (ER) retention sequence binding (Table 4).

**Table 4:** Gene ontology enrichment in muscle. All data (FDR < 5%) is shown.

Design	Category	Representation	GO-ID	Description
diet	CC	UNDER	GO:0005886	plasma membrane
		OVER	GO:0005747	mitochondrial respiratory chain complex I
		OVER	GO:0009328	phenylalanine-tRNA ligase complex
		OVER	GO:0005851	eukaryotic translation initiation factor 2B complex
	MF	UNDER	GO:0004888	transmembrane signaling receptor activity
		OVER	GO:0004826	phenylalanine-tRNA ligase activity
		OVER	GO:0051721	protein phosphatase 2A binding
	BP	OVER	GO:0006564	L-serine biosynthetic process
		OVER	GO:0006432	phenylalanyl-tRNA aminoacylation
		OVER	GO:0019243	methylglyoxal catabolic process to D-lactate via S-lactoyl-glutathione
		UNDER	GO:0007154	cell communication
		OVER	GO:0006417	regulation of translation
		OVER	GO:0022904	respiratory electron transport chain
		OVER	GO:0097272	ammonia homeostasis
		UNDER	GO:0044700	single organism signaling
		OVER	GO:0006119	oxidative phosphorylation
		OVER	GO:0009084	glutamine family amino acid biosynthetic process
enteritis	BP	OVER	GO:0006760	folic acid-containing compound metabolic process
		OVER	GO:0042558	pteridine-containing compound metabolic process
selection	MF	OVER	GO:0046923	ER retention sequence binding
strain	CC	OVER	GO:0022625	cytosolic large ribosomal subunit
		OVER	GO:0022627	cytosolic small ribosomal subunit
		OVER	GO:0005740	mitochondrial envelope
	MF	OVER	GO:0003735	structural constituent of ribosome
		OVER	GO:0044822	poly(A) RNA binding
		UNDER	GO:0004930	G-protein coupled receptor activity
		OVER	GO:0016992	lipoate synthase activity
	BP	OVER	GO:0000184	nuclear-transcribed mRNA catabolic process, nonsense-mediated decay
		OVER	GO:0006414	translational elongation
		OVER	GO:0006415	translational termination
		OVER	GO:0019083	viral transcription
		OVER	GO:0006614	SRP-dependent cotranslational protein targeting to membrane
		OVER	GO:0006413	translational initiation
		UNDER	GO:0007186	G-protein coupled receptor signaling pathway
		OVER	GO:0042255	ribosome assembly
		OVER	GO:0006099	tricarboxylic acid cycle
		OVER	GO:0042274	ribosomal small subunit biogenesis

### Co-regulation of genes

In order to assess the interplay between liver and muscle gene expression among the different comparisons, perturbation of all unique, significant DEGs between muscle and liver were mapped to KEGG pathway maps. We firstly identified 2899 unique transcripts, defined as those DEGs with unique gene ID’s between tissues. These transcripts were re-annotated using BLASTx and then assigned GO terms, Enzyme Codes and InterPro IDs where available. To assess the overall perturbation of the system between liver (the metabolically most-active tissue and largely tissue responsible for dietary changes) and muscle (growth during dietary substitution being the selection trait), these transcripts were then mapped to all KEGG-pathway reference pathway maps. By far the largest perturbed pathway was the map for purine metabolism, map00230. This map contains 152 unique sequences, out of the 2899 total perturbed sequences, and encompassed 25 enzymes (Table 5 and S1 Fig). This pathway was also the most significant when an assessment is made based on number of enzymes perturbed (Table 6). The second most perturbed pathway based on the number of sequences involved was thiamine metabolism. This pathway encompassed 117 sequences representing three enzymes. The overall themes of KEGG pathways that encompassed the most numbers of unique sequences was that of central and intermediary metabolism, drug and other foreign molecule metabolism, along with one immune pathway and one biosynthetic pathway (Table 5). However, when examining the data based on total number of enzymes perturbed in the system between muscle and liver, we can see a clear overall theme related to central and intermediary metabolism and associated biosynthetic pathways (Table 6).

**Table 5:** KEGG pathway reference maps perturbed combining unique muscle and liver significant differentially expressed genes, based on number of sequences. Cutoff for perturbation was set at a minimum of 20 sequences contained within a pathway.

KEGG map ID	KEGG pathway	Number of sequences	Number of enzymes
00230	Purine metabolism	152	25
00730	Thiamine metabolism	117	3
00627	Aminobenzoate degradation	39	3
00983	Drug metabolism—other enzymes	33	8
00620	Pyruvate metabolism	29	13
00010	Glycolysis / Gluconeogenesis	28	20
04660	T-cell receptor signaling pathway	26	2
00500	Starch and sucrose metabolism	26	13
00190	Oxidative phosphorylation	24	5
00020	Citrate cycle (TCA cycle)	23	12
00240	Pyrimidine metabolism	23	16
00980	Metabolism of xenobiotics by cytochrome P450	23	7
00040	Pentose and glucoronate interconversions	22	8
00982	Drug metabolism—cytochrome P450	22	6
00140	Steroid hormone biosynthesis	20	5
00380	Tryptophan metabolism	20	10

**Table 6:** KEGG pathway reference maps perturbed combining unique muscle and liver significant differentially expressed genes, based on number of enzymes. Cutoff for perturbation was set at a minimum of 10 enzymes contained within a pathway.

KEGG map ID	KEGG pathway	Number of sequences	Number of enzymes
00230	Purine metabolism	152	25
00010	Glycolysis / Gluconeogenesis	28	20
00240	Pyrimidine metabolism	23	16
00260	Glycine, serine and threonine metabolism	18	15
00620	Pyruvate metabolism	29	13
00500	Starch and sucrose metabolism	26	13
00270	Cysteine and methionine metabolism	18	13
00030	Pentose phosphate pathway	17	13
00020	Citrate cycle (TCA cycle)	23	12
00564	Glycerophospholipid metabolism	15	11
00250	Alanine, aspartate and glutamate metabolism	15	11
00052	Galactose metabolism	13	11
00380	Tryptophan metabolism	20	10
00970	Aminoacyl-tRNA biosynthesis	14	10

### Co-expression of genes in plant-diet tolerant selected trout

**Fig 5:**
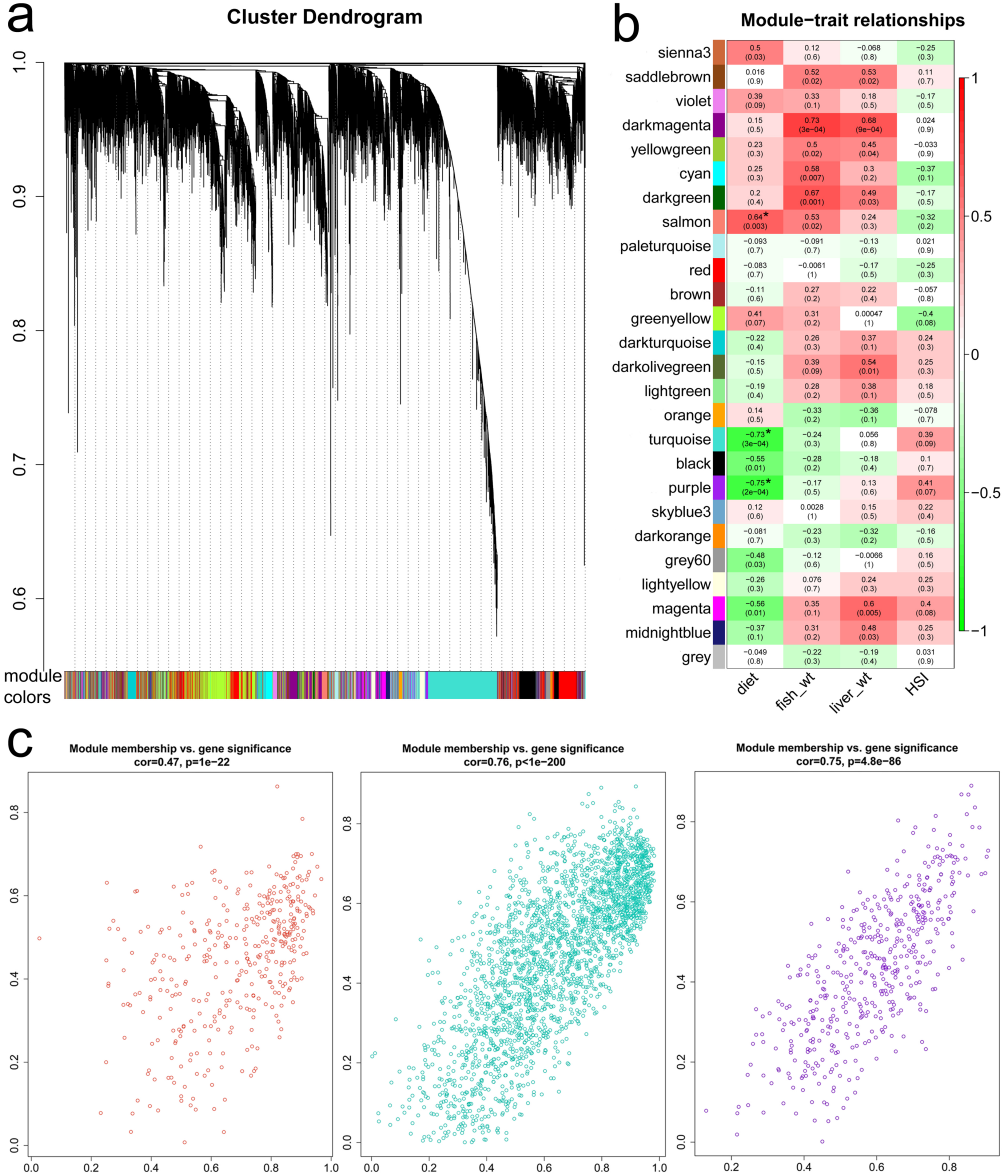
Weighted correlation network analysis among plant-diet tolerant selected fish fed either the FM diet (n = 10) or a 100% plant-protein replacement PM diet (n = 10), using RNA-seq data from liver tissues.

All ARS-KO selected fish (n = 20) were used to analyze liver RNA-seq data for any expression correlation patterns in fish fed different diets. As shown in Fig 5, a cluster dendrogram was created and genes were able to be assigned to various modules (Fig 5a). Continuing with the WGCNA, we included binary and continuous traits in the analyses to build module-trait relationships (Fig 5b). Included correlation traits were diet (either FM or PM), total fish weight, liver weight and hepatosomatic index (HSI). HSI is the ratio of liver weight to total weight. The index provides an indication on the status of energy reserves. Fish usually have a smaller liver in a poor environment and lower HSI is frequently seen in fish under stress. This can correlate to lower energy stores [43, 44]. We observed an inverse correlation with PM diet and HSI (Fig 5b). We also found three modules that were highly significantly (p ≤ 0.003) correlated with diet. These were selected for further analyses, as one major objective is identifying potential expression patterns, mechanistic genes and/or candidate genes for selection of plant-diet utilization without the fish being in a disease-state (i.e. without the confounder of enteritis). These significant modules included salmon, turquoise and purple (Fig 5b and 5c). Correlation plots of the three diet-significant modules examining module memberships plotted against gene significance show the data are well correlated and highly significant (Fig 5c). We identified 470, 388 and 2479 transcripts under the purple, salmon and turquoise modules, respectively.

GO analysis was performed on the genes underlying these three significant modules. As before, genes were mapped to GO terms and then two-sided Fisher’s exact test was used to assess significance between module genes and the rainbow trout transcriptome. No significance was found for genes underlying the purple module. While significant (FDR < 0.05) representation of ontology could not be found using Fisher’s exact test, upon further examination, genes underlying the purple module mapped largely to GO terms relating to purine metabolism. This was determined by assessing GO terms and building node charts in BLAST2GO, mapping graph nodes to various ontology levels, and then ultimately creating an annotation graph (graph statistics: sequence_filter = 30; node_score_alpha = 0.60) for each of the three GO categories (S2 Fig). Of the 470 transcripts identified under the purple module, 35 could be mapped to purine GO terms (GO:0001883-purine nucleoside binding; GO:0035639-purine ribonucleoside triphosphate binding; GO:0032555-purine ribonucleotide binding; GO:0017076 -purine nucleotide binding; GO:0032550-purine ribonucleoside binding). Additionally, 30 transcripts could be mapped to binding accessory functioning (GO:0016772-transferase activity transferring phosphorous containing groups).

GO analyses revealed genes underlying the salmon module are generally involved in gene regulation (S13 Table). There are 18 significant GO IDs including 6 from BP, 8 from CC and 4 from MF categories. There were both over- and under-represented terms. In the BP category, rRNA, tRNA mRNA processing as well as RNA methylation were all over-represented in this dataset, as was RNA binding and transferase activity under the MF category. In the CC category, the processome, pre-ribosome and terms associated with mRNA cleavage and polyadenylation specificity were also all significantly over-represented.

The turquoise module represents a large number of co-expressed genes. GO analysis revealed nearly 800 significant (FDR < 0.05) GO IDs (S14 Table). This dataset contains GO IDs from all three categories as well as both over- and under-represented terms.

Data includes 531 BP, 117 CC, and 143 MF category GO IDs. When assessing data manually by lowest FDR from each category, the most-significant ontologies are cytoplasm (CC; FDR = 2.1E^-52^), oxidation-reduction process (BP; FDR = 1.4E^-46^) and oxidoreductase activity (MF; FDR = 2.5E^-38^), all over-represented. In fact the top 100 most-significant ontologies are all over-represented. These top ontologies and this module appear to have a role in central and intermediary metabolism, as other highly significant GO IDs include general metabolic processes, small molecule, organonitrogen, peptide and carbohydrate metabolic processes as well as generation of precursor metabolites and energy (S14 Table).

### Candidate genes for trait selection based on plant-diet utilization

From the combined effort of differential gene expression, network and gene ontology analyses, a subset of genes were isolated as potential candidate genes for diet utilization (Table 7). These genes were selected from analyses using only ARS-KO selected fish—fish that perform well when fed a 100% plant protein replacement diet—but given different diets (Table 1). As such, our analysis has the potential to reveal genetic signatures important in diet utilization / plant-meal replacement without enteritis present, thus removing a confounding effect (noise) that may otherwise limit or muddle discovery. First to be selected a candidate, the top 30 hub genes from each module, i.e. those who have the highest degree of intramodular connectivity or co-expression, were collected. This list was filtered of any unknown, uncharacterized, hypothetical and structural proteins. Those remaining were labelled as hub (Table 7). We then selected the top 30 genes with the highest module membership, i.e. those with the highest eigengene-based connectivity (kME), and ranked them along with their Pearson correlation to diet to produce a weighted list of genes with the best (highest combined absolute value) network-based correlation (|kME + Cor|). This list was also filtered as before. We then sorted DEGs (S5 Table) from the diet comparison (Fig 1b) by highest significance using p-values adjusted for multiple comparisons and collected the top 1% by magnitude fold-change (DEG). Again we manually filtered this list as before. DEGs whose primary module membership was not one of the three significant to diet (salmon, purple or turquoise) were removed. All these data were ultimately combined to form a candidate gene list for potential surveillance, manipulation and/or selection of the plant-diet utilization trait, noting that genes compiled through different analyses may overlap as indicated (Table 7).

**Table 7:** Candidate gene list. Data listed along with each gene includes: fold-change values (FC) discovered through “diet” comparison gene expression analysis, illustrated in Fig 1; Module membership (MM) and diet-correlation values (Cor) from co-expression analysis. Significance column indicates method(s) of discovery to be considered a candidate gene.

Module	Gene symbol	Gene ID	Description	FC	MM	Cor	Significance
salmon	DNAJC2	C16211_c1_seq1_	DnaJ homolog subfamily C member 2	2.37	0.967	0.640	hub, |kME + Cor|, GO
	PPAN	C32677_c1_seq1_	Suppressor of SWI4 1 homolog	3.14	0.954	0.670	hub, |kME + Cor|, DEG, GO
	G3BP1	C14804_c0_seq1_	Ras GTPase-activating protein-binding protein 1	1.72	0.946	0.601	hub, |kME + Cor|, GO
	THUMPD1	C12905_c0_seq1_	THUMP domain-containing protein 1	2.45	0.940	0.558	hub, GO
	WDR55	C88818_c0_seq1_	WD repeat-containing protein 55	2.29	0.939	0.564	hub, GO
	FBL	C8062_c4_seq1_	rRNA 2'-O-methyltransferase fibrillarin	2.44	0.936	0.700	hub, |kME + Cor|, GO
	NOP58	C11232_c0_seq1_	Nucleolar protein 58	1.81	0.927	0.657	hub, |kME + Cor|, GO
	UTP3	C2534_c5_seq1_	Something about silencing protein 10	1.68	0.927	0.572	hub, GO
	DDX27	C18812_c3_seq1_	Probable ATP-dependent RNA helicase DDX27	2.49	0.923	0.649	hub, |kME + Cor|, GO
	PUM3	C34195_c0_seq1_	Pumilio homolog 3	1.66	0.919	0.551	hub, GO
	PRG4	C97287_c0_seq1_	Proteoglycan 4	2.98	0.820	0.862	|kME + Cor|, DEG
	UTP14A	C67976_c0_seq1_	U3 small nucleolar RNA-associated protein 14 homolog A	2.20	0.899	0.695	|kME + Cor|, GO
	GSPT2	C3298_c64_seq1_	Eukaryotic peptide chain release factor GTP-binding subunit ERF3B	1.83	0.889	0.700	|kME + Cor|, GO
	DDX56	C42805_c0_seq1_	Probable ATP-dependent RNA helicase DDX56	1.92	0.883	0.700	|kME + Cor|, GO
	DDX5	C2190_c0_seq1_	Probable ATP-dependent RNA helicase DDX5	1.75	0.887	0.693	|kME + Cor|, GO
	NOL8	C4288_c2_seq1_	Nucleolar protein 8	2.20	0.874	0.695	|kME + Cor|, GO
	MPHOSPH10	C44411_c0_seq1_	U3 small nucleolar ribonucleoprotein protein MPP10	2.27	0.880	0.681	|kME + Cor|, GO
	DNAJC21	C69765_c0_seq1_	DnaJ homolog subfamily C member 21	2.23	0.918	0.639	|kME + Cor|, GO
purple	EPCAM	C5086_c0_seq1_	Epithelial cell adhesion molecule	-1.64	0.907	-0.728	hub, |kME + Cor|
	LRRC8C	C63231_c0_seq1_	Volume-regulated anion channel subunit LRRC8C	-1.59	0.903	-0.713	hub, |kME + Cor|
	TXNIP	C5425_c0_seq1_	Thioredoxin-interacting protein	-3.22	0.883	-0.786	hub, |kME + Cor|, DEG
	KCNK5	C252072_c0_seq1_	Potassium channel subfamily K member 5	-2.04	0.871	-0.836	hub, |kME + Cor|
	G6PC	C1131_c10_seq1_	Glucose-6-phosphatase	-2.73	0.867	-0.576	hub
	slc25a36a	C38649_c0_seq1_	Solute carrier family 25 member 36-A	-2.77	0.861	-0.890	hub, |kME + Cor|, DEG
	IL17REL	C172972_c0_seq1_	Putative interleukin-17 receptor E-like	NA	0.853	-0.868	hub, |kME + Cor|
	FRK	C38093_c0_seq1_	Tyrosine-protein kinase FRK	-1.94	0.848	-0.819	hub, |kME + Cor|
	CLDN7	C8490_c0_seq1_	Claudin-7	-1.59	0.845	-0.708	hub, |kME + Cor|
	CTDSP2	C35474_c2_seq1_	C-terminal domain RNA pol II polypeptide A small phosphatase 2	NA	0.837	-0.456	hub
	SH2D3C	C38962_c1_seq1_	SH2 domain-containing protein 3C	-2.85	0.836	-0.740	hub, |kME + Cor|
	TRAF4	C72471_c1_seq1_	TNF receptor-associated factor 4	-1.81	0.833	-0.868	hub
	SGPP1	C21854_c1_seq1_	Sphingosine-1-phosphate phosphatase 1	-1.38	0.823	-0.705	hub, |kME + Cor|
	GMDS	C990_c3_seq1_	GDP-mannose 4,6 dehydratase	-1.95	0.813	-0.698	hub
	SNX9	C11879_c1_seq1_	Sorting nexin-9	-1.24	0.763	-0.815	|kME + Cor|
	NUPR1	C338_c259_seq1_	Nuclear protein 1	-2.35	0.802	-0.744	|kME + Cor|
	GCHFR	C8225_c0_seq1_	GTP cyclohydrolase 1 feedback regulatory protein	-1.27	0.803	-0.716	|kME + Cor|
	CDH17	C4892_c0_seq1_	Cadherin-17	-1.65	0.782	-0.730	|kME + Cor|
	PPP1R3B	C74190_c0_seq1_	Protein phosphatase 1 regulatory subunit 3B	-3.73	0.793	-0.714	DEG
turquoise	C8A	C4068_c0_seq1_	Complement component C8 alpha chain	-2.45	0.982	-0.720	hub, |kME + Cor|
	CD302	C9467_c0_seq1_	CD302 antigen	-2.13	0.981	-0.657	hub
	SPP2	C411_c37_seq1_	Secreted phosphoprotein 24	-2.73	0.979	-0.737	hub, |kME + Cor|
	EEF1G	C738_c2_seq1_	Elongation factor 1-gamma	-2.20	0.978	-0.677	hub
	HGD	C6448_c0_seq1_	Homogentisate 1,2-dioxygenase	-2.31	0.976	-0.725	hub, |kME + Cor|, GO
	SERPIND1	C1669_c0_seq1_	Heparin cofactor 2	-1.98	0.975	-0.674	hub
	SLC25A5	C241_c1_seq1_	ADP/ATP translocase 2	-2.22	0.975	-0.658	hub
	TMEM254	C6445_c0_seq1_	Transmembrane protein 254	-2.41	0.972	-0.697	hub
	MCFD2	C32034_c2_seq1_	Multiple coagulation factor deficiency protein 2	-2.57	0.972	-0.694	hub
	PC	C9567_c1_seq1_	Pyruvate carboxylase	-2.23	0.972	-0.696	hub, GO
	EIF5A2	C1423_c0_seq1_	Eukaryotic translation initiation factor 5A-2	-2.07	0.969	-0.662	hub
	PABPC1	C1010_c11_seq1_	Polyadenylate-binding protein 1	-2.19	0.969	-0.695	hub
	CLDN25	C18414_c0_seq1_	Putative claudin-25	-3.05	0.911	-0.835	|kME + Cor|
	MDH2	C1150_c0_seq1_	Malate dehydrogenase	-3.42	0.923	-0.816	|kME + Cor|, DEG, GO
	POR	C8317_c2_seq1_	NADPH—cytochrome P450 reductase	-3.06	0.936	-0.783	|kME + Cor|, GO
	HSD17B4	C19585_c0_seq1_	Peroxisomal multifunctional enzyme type 2	-2.31	0.958	-0.760	|kME + Cor|, GO
	FRIM	C160_c6_seq1_	Ferritin, middle subunit	-1.85	0.899	-0.815	|kME + Cor|, GO
	slc25a36a	C40704_c0_seq1_	Solute carrier family 25 member 36-A	-4.93	0.858	-0.855	|kME + Cor|, DEG
	ADAMTS13	C9888_c0_seq1_	Disintegrin and metalloproteinase with thrombospondin motifs 13	-2.03	0.872	-0.839	|kME + Cor|
	CP	C751_c0_seq1_	Ceruloplasmin	-2.28	0.965	-0.744	|kME + Cor|, GO
	ATP5F1	C1003_c0_seq1_	ATP synthase subunit beta	-2.89	0.928	-0.781	|kME + Cor|
	GLDC	C18716_c1_seq1_	Glycine dehydrogenase (decarboxylating)	-2.92	0.946	-0.762	|kME + Cor|, GO
	PROC	C4100_c0_seq1_	Vitamin K-dependent protein C	-2.26	0.958	-0.746	|kME + Cor|
	TSPAN1	C5350_c0_seq1_	Tetraspanin-1	-2.64	0.862	-0.841	|kME + Cor|
	bty	C93306_c0_seq1_	Protein similar to bloodthirsty / butyrophilin / TRIMs	-4.79	0.754	-0.874	DEG
	CES1	C37286_c0_seq1_	Liver carboxylesterase 1	-4.01	0.828	-0.778	DEG

The candidate gene list from this effort reveals a combined 63 transcripts to examine. This includes 18 from the salmon module, 19 from the purple module and 26 from the turquoise module. All transcripts except for two from the purple module were also found to be significant differentially regulated by read count-based DE analysis (Table 7 and S5 Table). Those two transcripts encode a putative interleukin-17 receptor E-like and a C-terminal domain RNA pol II polypeptide A small phosphatase 2. There is one gene within the list, solute carrier family 25 member 36-A, that was identified in two different modules but with different gene IDs. As would be expected, the two modules (purple and turquoise) where the gene with the same BLASTx identity (slc25a36a) was found both have the same diet-correlation relationship and the genes have the same direction of fold-change. This may represent different isoforms of the gene. Other intramodular transcripts were found that were members of a gene family such as the DDX helicases within the salmon module. There were three transcripts identified that only had support as a top DEG, including PPP1R3B, bty and CES1. These three belonged to the two negatively-correlated modules purple and turquoise. Overall, however, most candidate genes did have overlap in their method of discovery, either as a hub gene, a highly connected gene with high diet correlation and/or as a top differentially expressed gene (Table 7).

Even though this is a selected / shortened list of genes within each module, we selected / expected this list to the have highest intramodular connectivity and therefore biologically this could be indicative of genes that share a similar function. Thus those genes that could be assigned to GO groups were so indicated (Table 7), identified within each module through a one-sided (over-represented) Fisher’s exact test against the rainbow trout transcriptome. The purple module candidate genes showed no significant (FDR < 0.05) results. These results mirrored the full gene set from the purple module. The full gene set from the salmon module was heavily involved in gene regulation (S13 Table). For the salmon candidate gene set, we identified 24 over-represented GOs spanning all three categories, including three from MF, eight from BP and 13 from CC. Again as expected the overall theme is that of gene regulation, with all candidate genes under the salmon module assigned to significant GO group(s), except PRG4 (Table 7). The molecular functions of salmon candidate genes found were those of nucleic acid binding (GO:0003676), heterocyclic compound binding (GO:1901363) and organic cyclic compound binding (GO:0097159). For the turquoise candidate genes, there were a total of three significant over-represented GOs identified. They include ferroxidase activity (GO:0004322) and oxidoreductase activity oxidizing metal ions with oxygen as acceptor (GO:0016724) from the MF category as well as oxidation-reduction process (GO:0055114) from the BP category.

This candidate gene list was further assessed by mapping to EC numbers and determining which, if any, KEGG pathways could be identified containing multiple transcripts and/or enzymes. In the salmon module, four genes (DDX5, DDX27, DDX56 and GSPT2) were classified to have phosphatase (ec:3.6.1.15) activity and two genes (DDX27 and DDX56) are specific for adenylpyrophosphatase (ec:3.6.1.3) activity, which maps to and may play a role in purine (ko00230) and/or thiamine metabolism (ko00730). In the purple module, a protein-tyrosine kinase (FRK) and a phosphatase (CTDSP2) contributed to the identity of the T-cell receptor signalling pathway (ko04660). Two genes (PC and MDH2) classified for their carboxylase and dehydrogenase activity, respectively, contribute to both the citrate cycle (ko00020) and pyruvate metabolism (ko00620) from the turquoise module.

Several KEGG pathways were also identified in which only a single enzyme-encoding transcript from our candidate gene list contributed. The lysase GMDS (ec:4.2.1.47) mapped to the amino sugar, nucleotide sugar, fructose and mannose metabolism pathways (ko00520 and ko00051). G6PC contributed to the identification of glycolysis / gluconeogenesis, galactose, starch and sucrose metabolism pathways (ko00010, ko00052 and ko00500). In the turquoise module, CES1, a hydrolase and EEF1G, a transferase, play a role in xenobiotic metabolism pathways (ko00980 and ko00983). MDH2 contributes to the glyoxylate, dicarboxylate, methane, cysteine and methionine metabolic pathways (ko00630, ko00680 and ko00270). HGD, a 1,2-dioxygenase, contributes to both styrene degradation (ko00643) and tyrosine metabolism (ko00350). GLDC is classified as a aminomethyl-transferring dehydrogenase (ec:1.4.4.2) and contributes to glycine, serine and threonine metabolism (ko00260).

## Discussion

### Genetic selection relieves rainbow trout of enteritis fed a 100% plant-meal diet

Through genetic selection we developed a strain of rainbow trout that grows rapidly and tolerates diets containing protein from only plant sources. These findings have been previously reported [27, 28] and are further supported with histological findings in this study. Furthermore, earlier findings with similar diets found varying levels of detectable enteritis in non-selected fish but never in fish that had undergone more than four generations of selection. Research evaluating the relative aspects in the selected fish related to enhanced protein retention, changes in muscle cell development, and amino acid retention suggest changes occurring in liver, intestinal and muscle tissue biology. The current study was proposed to determine the effects of dietary selection in liver and muscle metabolism in trout after being fed an all plant protein feed. Earlier studies demonstrated the development of enteritis is readily detectable in non-selected trout after 12 weeks of feeding a similar PM diet [28]. While histological findings from the earlier study showed a greater level of tissue degradation in the non-selected fish, we were able to show a significant deterioration of intestinal tissue in non-selected fish in this study. Sample size was also lower for histological scoring and may account for some level of non-discrimination between each treatment. The previous study also demonstrated selected fish having improved growth on the plant-based feed and also improved protein retention efficiency [27]. To determine what adaptations relating to dietary utilization and growth are occurring in the ARS-KO line through selection and comparing this against non-selected fish with and without detectable enteritis, we evaluated all fish at 12-weeks when the non-selected fish were beginning to develop enteritis as detected by intestinal histology.

### Differentially expressed genes identify ontologies between trout strains given different diets

#### Liver

Analyzing liver expression in the non-selected trout fed either a FM or PM diet, we can compare and contrast DEGs as those reported in Atlantic salmon after 77 days on a replacement diet using microarray [45]. The authors found DEGs under the main categories of immune and stress response, cell proliferation and apoptosis, and protein metabolism. We identified similar categories, such as several related to a number of biosynthetic processes (Table 3 and S12 Table). These categories are also similar in the selection and strain group comparisons (Table 3). The difference lies within the diet comparison group, where ontologies of DEGs reveal gene regulation is much more pronounced here.

On the individual gene level, one similarity with salmon [45] includes expression of a gene encoding a proteasome, involved in protein turnover, found up-regulated in fish fed the PM diet (S7 Table). One such difference includes a gene encoding amine oxidase, involved in cell metabolic oxidation deamination, which we found to be down-regulated with PM diet. A plausible explanation could be species-specific effects, but we suspect different diet formulations could be responsible for variation observed. Overall with diet substitution in Atlantic salmon, we identified a similar number of DEGs in liver using non-selected fish. This is in great contrast to the number of DEGs identified when diet was substituted in the selected strain (Fig 4 and S5 Table), where we identified over 1K DEGs, which suggests that the selected trout are more responsive to plant diet substitution, having a potential greater capacity to metabolize the PM diet than non-selected, even as the magnitude of fold-change for many genes may not be as wide. This potential could also be evident as we see the greatest overlap of DEGs with diet/selection comparison (Fig 4). Both comparisons involve selected fish fed the PM diet but with different baseline samples for comparison (Fig 1b).

We also identified a transcript (C172972_c0_seq1) for interleukin-17 receptor E-like (IL17REL) from the enteritis comparison group (S7 Table) as the most down-regulated gene in the comparison. IL17REL proteins have been identified throughout chordates and the *il17rel* gene, along with neighboring genes, appears to be structurally conserved from fish to mammals [46]. Functionally, this gene likely binds specific IL-17 cytokines [46]. While IL17REL characterization in teleosts is lacking, recent evidence from both bony and cartilaginous fish show IL-17 receptors appear to function in mediating immune response as do their mammalian orthologs [47, 48]. Interestingly, a genome-wide association study and later whole-exome sequence analysis identified IL17REL [49, 50] as a major risk locus in humans for ulcerative colitis, and expression of IL-17 has been implicated in ulcerative colitis [51] / inflammatory bowel disease [52] and celiac disease [53]. This evidence suggests that the selected trout strain has potential as a biomedical model for human inflammatory bowel diseases.

#### Muscle

Overall, there were not as many DEGs identified in muscle as there were in liver tissues (Fig 4). The strain comparison identified the largest number of DEGs, which are largely involved in gene regulation (Table 4). GO for energy metabolism and oxidation of nutrients was most apparent in the diet comparison. This is interesting in that we see an inverse relationship with HSI and selected fish fed the PM diet (Fig 5). This interplay of energy stores and nutrient turnover in the muscle tissues of selected fish fed the PM diet could be important differences related to the utilization of different plant proteins. GO for immune responsiveness in muscle is not as pronounced as observed in Atlantic salmon fed a partial replacement diet [45].

Even as gene expression in muscle tissue is not as pronounced as in liver tissue during dietary alteration, the selected strain (ARS-KO) still grows twice as fast as parental lines and does so when fed a high-soy, all plant-protein feed [27]. Thus additional examination of muscle tissue outside of mRNA may further explain physiological differences of fish strain to PM diet. For instance, phenotypic variation at the nucleotide level (SNPs) may explain differences of selective muscle growth to PM diets. In zebrafish, growth-associated SNP variation was noted in the aminoacyl-tRNA biosynthesis pathway when fish were fed a plant-protein diet [54]. We observed that the aminoacylation GO biological process is significantly enriched in muscle (Table 4) and that aminoacyl-tRNA biosynthesis KEGG pathway is highly perturbed at the systems level (Table 6). Also, dietary-induced epigenetic modifications to skeletal muscle growth are indicated in a variety of food animal and livestock species as are regulatory RNAs [55]. We now understand certain regulatory RNAs play a role in fish muscle growth, feed intake and nutrient metabolism [56], which may be indicated in our functional analyses (GO:0006417). As ER retention sequence binding (GO:0046923) is the only enriched functional category in the muscle selection comparison (Table 4), it is also highly likely that variation in muscle due to selective pressures under PM diet manifests at the protein level, which remains to be further explored with this trout model.

### Synergistic effects of gene perturbation combining liver and muscle expression

Our trout breeding program is based upon growth and feed efficiency selection with tolerance to PM diets (ARS-KO strain). Therefore, we were interested in determining any gene expression signatures that may be suppressed or amplified when comparing DEGs from both muscle and liver tissues. We found that when combining tissues for analyses, the pathway for purine metabolism is extraordinarily perturbed (S1 Fig) in both the number of transcripts and enzymes detected under this systems approach given all unique DEGs, regardless of strain or diet type (Tables 5 and 6). One possibility for this perturbation could be the amount of nucleotides in foodstuffs and additives selected for aquafeeds [57], since meat and seafood products generally have higher purine content than plant products. However, soybean is the major plant ingredient in the PM diet (Table 1), which is one of the more purine-rich plantstuffs [58]. We did not find differential expression of urate oxidase mRNAs in our datasets, one of the genes found responsive to changes in purines in the diets of Atlantic salmon [59].

Perturbation of purine metabolism at the systems level may also be due to amplified effects of nucleotide differences on innate immunity, as we observe a moderate number of transcripts contributing to T-cell signalling pathway in both liver and muscle (Table 5). Indeed in mammalian physiology, adenosine released at injured and inflamed sites plays a central role in the regulation of inflammatory responses and in limiting inflammatory tissue destruction [60, 61]. Whereas early inflammatory signaling favors a transition from neutrophil infiltration to macrophage recruitment, in later stages adenosine contributes to resolving inflammation both by down-regulating macrophage activation and by progressing T-cell responses [60, 61]. Some anti-inflammatory and immuno-modulating drugs act, at least in part, by decreasing intracellular adenosine 5’-triphosphate (ATP) concentrations and increasing extracellular adenosine levels [61, 62]. Exosome enzymatic activity has also been indicated in the production of extracellular adenosine, related to T-cell regulation [63]. Among the greatest CC GOs in our datasets are the cytosol and extracellular exosome within the diet comparison (Table 3). It is thus highly plausible in our experimental model, where both purine metabolism (highly) and T-cell signalling (slightly) are perturbed, that we are observing responsiveness to quelling tissue disruption and inflammation rather than simply a difference in metabolic demands due to some dietary disparity. Genetic selection in trout for diet utilization may have shifted this interplay between diet and nonspecific immunity. Further examination on relationship of purine metabolic homeostasis versus the level of inflammation is warranted based on the observations here, particularly in our enteritis-free trout model at the site of inflammation (DI).

Other major pathways perturbed among tissues are ones that might be expected, relating to many central metabolic processes and the production of energy along with responsiveness to foreign substances (Tables 5 and 6).

### Enteritis-free trout strain reared on PM diet reveals candidate genes for dietary utilization

Our candidate list reveals a set of genes from three modules highly correlated with diet substitution using plant-diet tolerant trout (Table 7). The positively-correlated module (salmon) contains genes involved in RNA-binding, RNA-processing and RNA methylation (S13 Table). Since modules identified have high correlations, albeit direct or inverse with diet, it is likely that some RNA regulation (genes from the salmon module) is affecting genes of the other significant modules. For instance, elongation factors can play a role in mRNA turnover and processing of polypeptides through delivery of amino acids to ribosomal complexes. We see that elongation factors are down-regulated (and negatively-correlated) with the PM diet. Based on KEGG mapping, candidate enzymes encoded from genes in the salmon module may play a role in purine and/or thiamine metabolism. Regulation of both nucleotides and essential nutrients might be one way in which selection has affected plant diet tolerance. In terms of enteritis, alternative mRNA processing [64], noncoding RNAs [65] and epigenetic effects [66] are also strongly indicated in inflammatory bowel diseases. The ability of the selected fish to quell enteritis could also be indicative of some of the strong regulatory signatures we see here, that are positively correlated to PM diet and the salmon module (S13 Table).

Candidate genes under the purple and turquoise modules are negatively-correlated with the PM diet. In GO analyses of genes underlying the complete purple module, we find a high number of GO terms associated with purine metabolism (S2 Fig). Along with a positive correlation to potential regulation of metabolism from genes in the salmon module, this provides more evidence for the candidate genes’ involvement in (regulation of) purine metabolism as important in long-term plant diet tolerance. Further evidence supporting nucleotide importance is that one of the genes, slc25a36, in mammals is involved in pyrimidine transport [67]. The interconnectedness and role of this candidate gene in trout dietary tolerance requires further study as purine signals are by far the most prevalent in this study, although in general, DE of genes involved in nucleotide synthesis are often paralleled by immune responsive genes in salmonids [68]. Even as we are examining liver co-expression, since we were most determined to find nutrigenomic signatures outside of enteritis, we still observed that the purple module shows KEGG pathways involved in T-cell receptor signalling. In addition, on the 100% plant protein replacement diet fed to tolerant selected fish with no enteritis, we observed some related down-regulated genes that have previously been up-regulated on plant replacement diets in salmonids. For instance, after 8 h on a plant-diet, Panserat and colleagues [12] found that a thioredoxin-like protein gene and sorting nexins, similar to what was found in this study, were up-regulated in trout liver. Although these may be different isoforms of genes discovered here, there is evidence that selection, long-term tolerance and a difference in gene regulation could all play a role in our observations. For candidate genes under the turquoise module (Table 7), we observe that two genes encode enzymes (PC and MDH2) classified for their carboxylase and dehydrogenase activity, respectively, that contribute to both the citrate cycle and pyruvate metabolism. In addition we see ferroxidase activity and oxidoreductase activity oxidizing metal ions GOs from these candidate genes. Together these genes have been shown to be involved with modulating humoral immune response, protein folding and cell survival [69–71]. Overall this suggests that utilization of the PM diet involves synergistic effects of genes involved with maintaining cell and tissue health at multiple stages, from transcriptional regulation and protein modification to cell signalling and related responses.

Previous studies have shown T-cell involvement in soybean meal diet-induced intestinal inflammation in salmonids [19, 72, 73]. Lilleeng and colleagues [72] found that expression of T-cell markers was depressed after early exposure of Atlantic salmon to a soybean meal diet. We observed a tyrosine kinase (FRK) and a phosphatase (CTDSP2) contributing to the identity of the T-cell receptor signalling KEGG pathway along with an interleukin receptor (IL17REL) were identified as having an inverse relationship to PM diet (Table 7). FRK was also identified as negatively-regulated through differential gene expression analysis. Interestingly, IL17REL and CTDSP2 were the only two genes in our candidate list that were not significant DEGs between the diet comparison (Fig 1b). However, in the chronic enteritis comparison (S7 Table), IL17REL is the largest (by magnitude) down-regulated gene given PM diet treatment for 12-weeks, a gene which has been strongly associated to human ulcerative colitis [49]. These data suggest that regulation of T-cell associated activity is not only important in short-term exposure to PM diet but also in the longer-term, with presence of enteritis being the main driver over genotype. This is supported by the fact that IL17REL, CTDSP2 and FRK are not DEGs in the selection comparison (Fig 1b). This also reveals that the magnitude of fold-change may not be the only key for long-term T-cell (receptor) activity under PM-induced enteritis, but rather feedback mechanisms, the interplay with other co-expressors or, in the presence / up-regulation of various gene regulators (salmon module), transcriptional or post-transcriptional regulation all could play a role. These could contribute to the ways in which ARS-KO fish, free of enteritis on PM diet, suppresses inflammation under what would be (in the non-selected fish) chronic enteritis by 12-weeks’ time. It is also possible that through selection trout strains may be having different autoimmune or other detoxification responsiveness given different diets, as perturbation of muscle and liver tissues combined reveals both T-cell receptor signalling and metabolism of xenobiotics by cytochrome P450 as key pathways within the system (Table 5). Indeed perturbation of genes underlying xenobiotic metabolism have been observed in many cases of diet modifications in salmonid and other marine fish aquaculture [24, 74, 75]; in addition, a gene for cytochrome P450 reductase was identified as a candidate in this effort. As the major objective of this study was focused towards nutrigenomics, further functional analyses at the site of inflammation (distal intestine) using ARS-KO selected trout may reveal further important signatures of enteritis, especially given these immune-related signatures in liver.

Candidate genes that are found linked to the trait of plant-protein utilization in carnivorous fish would be highly advantageous for use in aquaculture, especially given the rising costs and lack of availability of FM [5]. Additionally, the identification of variation explaining phenotypic effects is especially valuable for traits that are difficult and/or expensive to evaluate [76], such as those requiring extended rearing periods. In our rainbow trout model, candidate gene data can provide additional information about the biology behind our trait as well as reduce costs in terms of number of resources needed for selection when used as a screening tool. This information also has the potential to be transferred to other teleosts, most specifically other salmonids, in order to increase genetic gains through marker-assisted or genomic selection [76, 77].

## Conclusions

The reformulation of aquafeeds along with the advancement of breeding programs with species adaptive to replacement diets is crucial to the success of sustainable aquaculture. After over a decade of genetic selection, we developed a strain of rainbow trout that performs well over long-term rearing when fed a high-soy, all plant-protein feed without developing enteritis. Since growth and feed efficiency along with diet-tolerance were major selection foci, we examined gene expression by RNA sequencing muscle and liver tissues of selected and non-selected trout strains fed either a fishmeal-based control feed or an all plant-protein feed. Genes identified in this study can be added to the growing list of those associated with diet tolerance and utilization. Among the tissues and strains, thiamine and especially purine metabolism is highly perturbed. Systems analysis among the tissues tested reveals that the interplay between selection for growth, dietary utilization and tolerance may have implications in nonspecific immunity as well. Using an integrative nutrigenomic approach of read-count differential expression, co-expression and other functional analyses, candidate genes that may play major roles in plant-diet utilization were found as these genes were identified in fish in the absence of enteritis. In addition, a major risk locus in ulcerative colitis, IL17REL was identified through these efforts and expression of IL-17 has been indicated in ulcerative colitis and celiac disease; as such, rainbow trout selected for plant-diet tolerance may have added utility as a potential biomedical model for human inflammatory bowel diseases.

## Supporting information

S1 Fig PDF image of purine metabolism KEGG pathway, colored by enzyme. (PDF)

S2 Fig PDF image of GO pie chart based on purple module from WCGNA. (PDF)

S1 Table Excel file of trait measurements used in WGCNA. (XLSX)

S2 Table Excel file of FPKM values used in WGCNA. (XLSX)

S3 Table Excel file of primer information for TaqMan assays used in RT-qPCR. (XLSX)

S4 Table Excel file of DEGs from liver in the “selection” comparison. (XLSX)

S5 Table Excel file of DEGs from liver in the “diet” comparison. (XLSX)

S6 Table Excel file of DEGs from liver in the “strain” comparison. (XLSX)

S7 Table Excel file of DEGs from liver in the “enteritis” comparison. (XLSX)

S8 Table Excel file of DEGs from muscle in the “selection” comparison. (XLSX)

S9 Table Excel file of DEGs from muscle in the “diet” comparison. (XLSX)

S10 Table Excel file of DEGs from muscle in the “strain” comparison. (XLSX)

S11 Table Excel file of DEGs from muscle in the “enteritis” comparison. (XLSX)

S12 Table Excel file of the complete GO analysis of liver based on DEGs. (XLSX)

S13 Table Excel file of Fisher’s exact test results of salmon module from WCGNA. (XLSX)

S14 Table Excel file of Fisher’s exact test results of turquoise module from WCGNA. (XLSX)
