# Supplementary figures and images for: Integrative functional analyses using rainbow trout selected for tolerance to plant diets reveal nutrigenomic signatures for soy utilization without the concurrence of enteritis
Source: PLoS One. 2017 Jul 19;12(7):e0180972. doi: 10.1371/journal.pone.0180972 (PMC5517010; doi:10.1371/journal.pone.0180972)

# PURINE METABOLISM

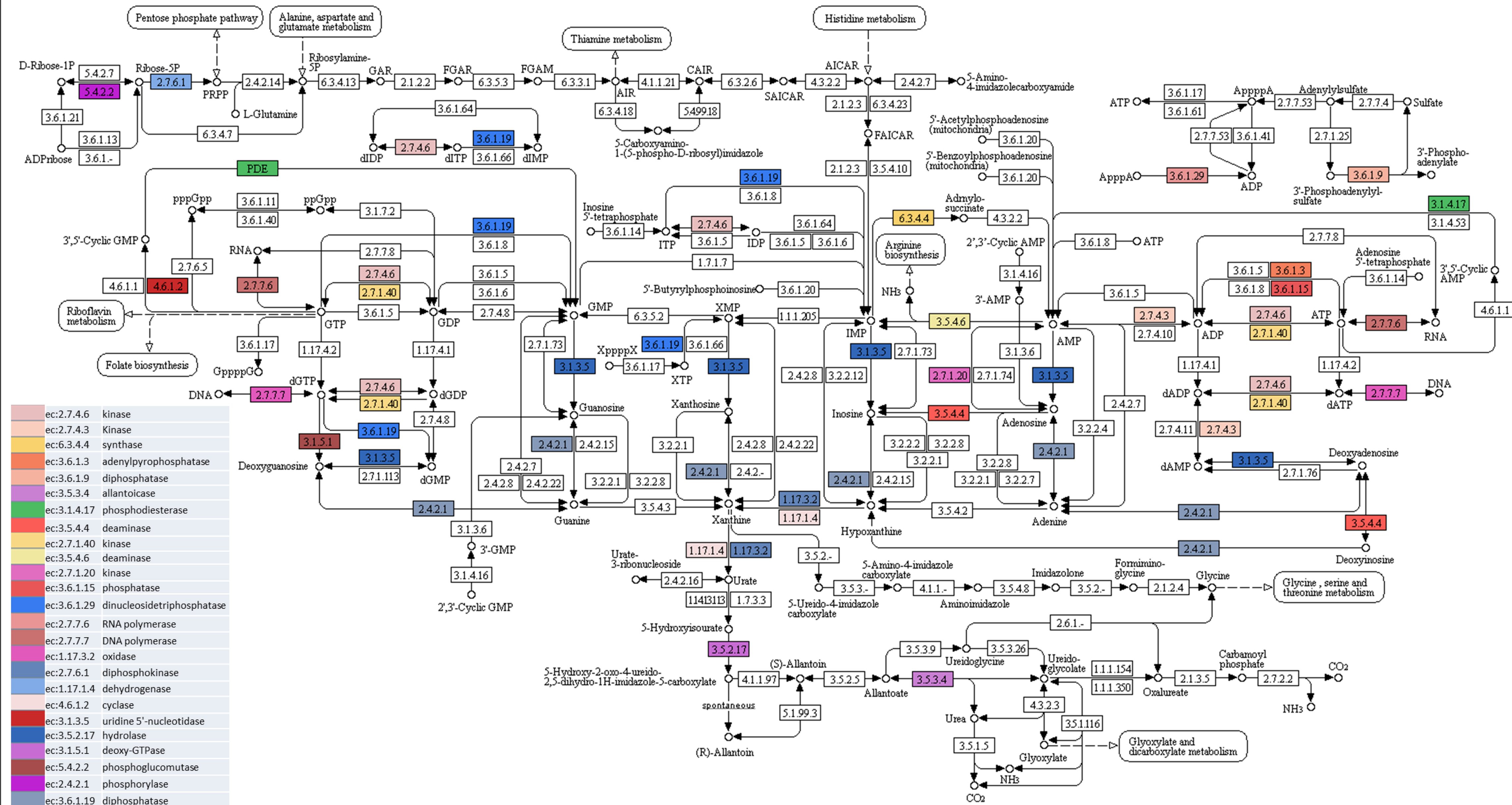

Supplement: S1 Fig — (PDF) [file pone.0180972.s001.pdf]
